# TRANSITIONAL CARE TRENDS IN BENEFICIARIES WITH HEART FAILURE AND DEMENTIA

**DOI:** 10.1093/geroni/igae098.2597

**Published:** 2024-12-31

**Authors:** Thomas Bayer, Hiren Varma, Peter Hollmann, Pedro Gozalo

**Affiliations:** Providence VA Medical Center, Providence, Rhode Island, United States; Brown University, Providence, Rhode Island, United States; Brown University Medical School, Providence, Rhode Island, United States; Brown University, Providence, Rhode Island, United States

## Abstract

Medicare Transitional Care Management (TCM) has grown since its introduction in 2013, but we know little about utilization among beneficiaries with dementia (BWD) undergoing the complex care transition from hospital to skilled nursing facility to home (hospital-SNF-home). We studied trends in TCM among BWD discharged to various care settings after heart failure hospitalization, given heart failure’s high readmission risk and amenability to care management. We selected a sample of BWD heart failure hospitalizations in years 2013-2018 using the Chronic Conditions Warehouse algorithm for Alzheimer’s Disease and Related Dementias and principal diagnostic codes of hospital claims. Next, we excluded all except hospital-home and hospital-SNF-home discharges. We then fit a linear regression with TCM as the dependent variable and discharge year, discharge type, interaction of year and discharge type, race, age, dual status, and sex as predictors. TCM after hospital-home discharges grew from 521 (5.3%) of the 9,930 eligible discharges in 2013 to 2,015 (15.9%) of the 12,706 eligible discharges in 2018. In contrast, TCM after hospital-SNF-home discharges grew from 80 (4.0%) of the 1,990 eligible discharges in 2013 to 207 (9.9%) of the 2,095 eligible discharges in 2018. The P-value for the interaction between year and discharge type was <.001. The proportion of BWD utilizing TCM after heart failure hospitalization grew less among hospital-SNF-home discharges than hospital-home discharges. Further study is needed to understand why TCM utilization in hospital-SNF-home discharges has lagged behind hospital-home discharges and to estimate TCM’s effect on the success of discharge from SNF to home.

